# Genomic Differences Between Two *Fusarium oxysporum* Formae Speciales Causing Root Rot in Cucumber

**DOI:** 10.3390/jof11020140

**Published:** 2025-02-12

**Authors:** Ernest Nailevich Komissarov, Roderic Gilles Claret Diabankana, Inna Abdeeva, Daniel Mawuena Afordoanyi, Sergey Vladimirovich Gudkov, Ekaterina Mikhailovna Dvorianinova, Sergey Alexandrovich Bruskin, Alexey Alexandrovich Dmitriev, Shamil Zavdatovich Validov

**Affiliations:** 1 Laboratory of Molecular Genetics and Microbiology Methods, Kazan Scientific Center of the Russian Academy of Sciences, 420111 Kazan, Russia; d.afordoanyi@knc.ru (D.M.A.); sh.validov@knc.ru (S.Z.V.); 2 Vavilov Institute of General Genetics, Russian Academy of Sciences, 119991 Moscow, Russia; insaz@vigg.ru (I.A.); brouskin@vigg.ru (S.A.B.); 3 Prokhorov General Physics Institute of Russian Academy of Sciences, 119991 Moscow, Russia; s_makariy@rambler.ru; 4 Institute of Biology and Biomedicine, Lobachevsky State University of Nizhny Novgorod, 603022 Nizhny Novgorod, Russia; 5 Engelhardt Institute of Molecular Biology, Russian Academy of Sciences, 119991 Moscow, Russia; dvorianinova.em@phystech.edu (E.M.D.); alex_245@mail.ru (A.A.D.)

**Keywords:** *Fusarium oxysporum*, *radicis-lycopersici*, *radicis-cucumerinum*, root rot, *SIX* genes, *mimp*, effector profile, pathogenicity, disease index, effector genes

## Abstract

The host specificity of *Fusarium oxysporum* (*Fox*) formae speciales has been reported to be linked to effector proteins known as Secreted in Xylem (SIX). These genes are associated with the non-autonomous mobile element miniature impala (*mimp*), normally distributed on the accessory chromosomes. The pattern of *mimp* associated with effector genes has been used to predict candidate effector profiles which characterize *Fox* formae speciales. In this study, we demonstrate the pathogenicity of strains *Fusarium oxysporum* f.sp. *radicis-lycopersici* (*Forl*) ZUM2407 and *Fusarium oxysporum* f.sp. *radicis-cucumerinum* (*Forc*) V03-2g in a common host plant (cucumber) and compare their genomes. The *Forl* ZUM2407 genome lacks *SIX* genes and their homologs, in contrast to *Forc* V03-2g. We predicted the total number of *mimp* elements in the genome of *Forl* ZUM2407 to be three-fold less than that of *Forc* V03-2g (10 and 36 copies, respectively). The *mimp* distribution pattern in *Forl* ZUM2407 was completely different from that present in *Forc* V03-2g. Candidate effector profile analysis did not predict that *Forl* ZUM2407 was able to infect cucumber plants like *Forc* V03-2g. Therefore, we assume that *Forl* ZUM2407 has a different type of genome organization associated with pathogenicity, whose effector profile cannot be described using the *mimp*-based approach.

## 1. Introduction

The *Fusarium oxysporum* species complex (FOSC) is a group of soil mycelial fungi consisting of both non-pathogenic (saprophytes and endophytes) and pathogenic forms that cause foot and root rot and wilt in a variety of economically valuable crops [1]. The pathogenic strains of the FOSC exhibit the ability to infect one or a group of closely related plant species [2]. Based on this specificity to their host plants, strains of *Fusarium oxysporum* (*Fox*) are distinguished by their formae speciales, which are strains of a given species that can infect the same groups of plants. Currently, there are 106 reliably described *Fox* formae speciales and 37 less accurately characterized ones, indicating a huge diversity among the pathogens of the FOSC [3] despite the absence of sexual reproduction in this species [4]. With the help of comparative genomics, a large number of defined genomes called core chromosomes have been predicted to be present in all FOSC strains, with pathogenic strains having additional chromosomes (supernumerary/accessory) [5]. The varying size of these additional chromosomes is mainly responsible for the significant variation in the total genome size among different *Fox* strains [5]. The transfer of additional chromosomes to non-pathogenic strains of *Fox* leads to the acquisition of pathogenicity in initially non-pathogenic strains, and probably a change in the range of host plants, explaining the existing intraspecific diversity and polyphyletic origin of most formae speciales [6,7,8,9]. Since horizontal gene transfer on additional chromosomes is probably an important process for the development/evolution of formae speciales [10,11], close attention has been paid to the study of the additional genomes of these species, which are characterized by a low gene density and a high density of genetic repeats. The most studied genome is the genome of the vascular pathogen *Fusarium oxysporum* f.sp. *lycopersici* (*Fol*), which causes wilt in tomatoes [12]. The secretion of effector proteins (Secreted in Xylem (SIX)) in the xylem sap of host plants by *Fol* is well documented to play a crucial role in bypassing the plant defense system and colonizing host plants [13,14]. When the sequences of the accessory genome were studied in more detail, it was found that sequences of the non-autonomous mobile genetic element miniature impala (*mimp*) were present in the promoter region of all known *SIX* genes [15,16]. This observation led to the hypothesis that the association of *mimp* elements with SIX genes is a unique characteristic for the localization of effector genes in *Fox*. By applying this hypothesis, additional genes were identified and subsequently confirmed to be expressed in the xylem sap, expanding the known repertoire of *SIX* genes [15]. This approach has been extended to other formae speciales of *Fusarium oxysporum* to identify candidate effector proteins that contribute to their host specificity and pathogenicity. The pathogenicity of *Fol* strains in different varieties of tomato plants has been documented to be based on avirulent genes (such as *SIX*), which allows the classification of *Fol* strains into different races based on their presence in the genome [15,17].

Several works emphasize the association of *SIX* genes with root rot development [18,19,20]. Additionally, the necessity of an accessory chromosome carrying *SIX* genes in *Fusarium oxysporum* f.sp. *radicis-cucumerinum* (*Forc*) for foot and root rot development in *Cucurbitaceae* plants was demonstrated, taking the *Forc*016 strain as an example [18]. Consequently, this chromosome was named RC, indicating its role in pathogenesis for *radicis-cucumerinum* formae speciales [18]. The presence of *SIX* genes in *Fusarium oxysporum* f.sp. *radicis-lycopersici (Forl)* has not yet been reported and has been suggested to be absent in the genomes of this forma specialis [5]. In contrast to *SIX* genes, there may be other genetic determinants or effectors responsible for the ability of *Forl* to cause root rot. Therefore, this work aims to compare the aggressiveness of the phytopathogens *Forl* ZUM2407 and *Forc* V03-2g in causing root rot in tomato and cucumber plants and analyze their genomes to identify gene sets encoding candidate effector proteins based on *mimp* transposon distributions, which are frequently used for host specification in *Fox*.

## 2. Materials and Methods

### 2.1. Fungal Strains

*Fusarium oxysporum* f.sp. *radicis-cucumerinum* (*Forc*) V03-2g used in this study was isolated from infected greenhouse cucumber plants in the All-Russian Research Institute of Agricultural Microbiology (Saint-Petersburg-Pushkin, Russia). *Fusarium oxysporum* f.sp. *radicis-lycopersici* (*Forl*) ZUM2407 used in this study was isolated from infected tomato plants by the DLO Research Institute for Plant Protection (Wageningen, The Netherlands). The pathogenic strains *Forc* V03-2g and *Forl* ZUM2407, which cause foot and root rot in cucumber (*Cucumis sativus* L.) and tomato (*Solanum lycopersicum* L.), respectively [21,22], were provided by the laboratory of Molecular Genetics and Microbiology Methods.

### 2.2. Plant Varieties

The tomato varieties “Belyi Naliv” (Agrofirm POISK, Moscow, Russia) and “Mos-kvich” (Agrofirm Sedek, Moscow) and the cucumber varieties “Palchik” (Volzhskiy sad, Republic of Tatarstan, Russia) and “Parizhskiy Kornishon” (Agrofirm POISK, Moscow, Russia), which are susceptible to foot and root rot caused by *Forl* ZUM2407 and *Forc* V03-2g, respectively, used in this study were purchased from registered seed growers.

### 2.3. Disease Assays, Growth Conditions, and Seed Inoculation

The pathogenicity test was performed under laboratory conditions. Tomato and cucumber seeds were sterilized as described by Simons et al. [23]. After sterilization, cucumber and tomato seeds were placed in a Petri dish moist chamber and incubated at 25 °C for 1 and 3 days, respectively, for pre-germination. Fungal spores were prepared from a 5-day-old culture of *Forl* ZUM2407 and *Forc* V03-2g grown in potato dextrose medium (PDA) [(potato broth—200 g/L), dextrose—2% (m/v)] and filtered through sterilized cotton wool. The suspension was centrifuged at 4000 rpm for 2 min using a microcentrifuge Centrifuge 5810 R (Eppendorf, Hamburg, Germany). After centrifugation, the resulting pellet was resuspended in saline solution [0.9% (m/v) NaCl]. Spores were counted using a hemocytometer. Pre-germinated seeds were placed in the spore suspension for 30 min and sown in plastic pots with a size of 37 × 16 × 15 cm (length × width × height) containing sterile sand amended with plant nutrient solution [24] in a ratio of 1:10 (*v*/*v*). Pots were sealed with polyethylene film to prevent evaporation. Pots were placed in controlled-climate chambers (Phytotron Ltd., Moscow, Russia) under the following parameters: humidity of 60%, and a day–night light cycle of 12/12 h at 25 °C and 21 °C, respectively. For statistical analysis, the experiment was performed thrice. Each group was maintained in three replicates which contained 15 tomato and 6 cucumber plants (Figures S1 and S2). Tomato and cucumber plants were cultivated for up to 1 week and 2 weeks, respectively. After cultivation, the plants were removed from the pot and washed with running tap water. The disease severity was evaluated using foot and root rot symptoms on a scale from 0 to 4 (0–4), as previously described by Validov et al. [22], with modifications for damping-off assessment: 0—healthy plants; 1—plants with small lesions (weak root browning/weak cotyledon chlorosis); 2—plants with developed lesions (clearly visible symptoms of root rot in the area of the root neck and main root/yellowed cotyledons); 3—plants with large lesions (root rot ascends along the hypocotyl to cotyledons/wilted and browned cotyledons); 4—dead plants (Figures S6a–d). The disease index (DI) was calculated using the following formula [25]:

$$\mathrm{DI}=\left(\sum \frac{\mathrm{ab}}{\mathrm{NK}}\right)\times 100\%$$

where a—the number of plants with different disease severity scales; b—disease severity scale; N—total number of plants; and K—the maximum disease severity. Statistical data analysis was performed using the statistical program OriginLab Pro SR1 b9.5.1.195 (OriginLab Corp., Northampton, MA, USA). The disease index between groups was analyzed using a one-way ANOVA test (*p* < 0.05).

Furthermore, the infected plants were subsequently used for the *Fusarium* strains’ re-isolation and genetic comparison with *Forl* ZUM2407 and *Forc* V03-2g.

### 2.4. Pathogen Re-Isolation from Affected Plant Tissues

Pathogen re-isolation was performed using the spread plate method. For this purpose, affected plant areas were surface-sterilized according to Simons et al. [23] and placed on potato dextrose agar (PDA). Grown mycelium was resuspended in saline solution, serially diluted to 10^−3^, and replated on PDA. Single colonies were randomly picked and compared with *Forl* ZUM2407 and *Forc* V03-2g using BOX-PCR analysis.

### 2.5. Molecular, Genetic, and Bioinformatic Analysis

#### 2.5.1. DNA Isolation

DNA isolation was performed on 1 g of mycelium ground in liquid nitrogen as described in Krasnov et al. [26]. Multiple rounds of chloroform extraction and precipitation, were performed, as well as treatment with RNase A and proteinase K. To prepare sequencing libraries, super-long DNA was removed from the first elution after the step of column purification. The remaining DNA pool was further used. The DNA quality and concentration were evaluated on a NanoDrop 2000C spectrophotometer (Thermo Fisher Scientific, Waltham, MA, USA) and a Qubit 4.0 fluorometer (Thermo Fisher Scientific, Waltham, MA, USA) and by agarose gel (1%) electrophoresis.

#### 2.5.2. BOX-PCR Analysis

BOX-PCR was performed in a 20 µL volume containing 2 µL of 10× PCR buffer, 0.4 µL of dNTP mixture (10 µM), 0.4 µL of 10 µM BOXAIR (5′-CTACGGCAAGGCGACGCTGACG-3′), 1.0 µL of DNA template (100 ng), 0.4 µL of Taq DNA polymerase (5 U), and nuclease-free water. Amplification was performed using a T100 thermocycler (Bio-Rad, Hercules, CA, USA) under the following conditions: initial denaturation at 95 °C for 2 min; 30 cycles of 94 °C for 30 s and 58 °C for 30 s; and 72 °C for 8 min. The final cycle was extended to 72 °C for 10 min. The DNA fragments were detected via 1.5% agarose gel electrophoresis and visualized on a Gel Doc EZ Imager with Image Lab 6.0 software (Bio-Rad, Hercules, CA, USA).

#### 2.5.3. Genome Sequencing, Assembly, and Analysis

For genomic sequencing, DNA fragments less than 10 kb were removed from the DNA samples using the Short Read Eliminator Kit (PacBio, Menlo Park, CA, USA) according to the manufacturer’s instructions. DNA library preparation was performed using the SQK-LSK109 Ligation Sequencing Kit (ONT, Oxford, UK) for 1D genomic DNA sequencing. Sequencing was performed on MinION (ONT, Oxford, UK) with a FLO-MIN-106 R9.4.1 flow cell (ONT, Oxford, UK). Raw fast5 files generated by MinION sequencing were basecalled with a super-accuracy flip-flop algorithm (dna_r9.4.1_450bps_sup.cfg configuration file) with a min qscore of 10 and further converted to fastq reads using the ONT basecaller Guppy v.6.3.7. Adapters were then trimmed using Porechop v.0.2.4. (https://github.com/rrwick/Porechop, accessed on 13 March 2023).

Genome assembly was carried out using Canu v.2.2 with default parameters and the genome size set to 70 Mb [27]. The obtained assemblies were polished with Nanopore reads using Racon v.1.5.0 (2 iterations) [28] and Medaka v.1.7.0 (https://github.com/nanoporetech/medaka, accessed on 13 March 2023).

Repeat elements were identified using RepeatModeler v. 2.0.4 [29] and RepeatMasker v. 4.1.5 [30]. Gene prediction was carried out on the masked genome using Maker v. 2.31.11 [31] and the protein sequences of the 3 most closely related as well as well-annotated FOSC strains (Forc016, Fol4287, Fo47; accession numbers: ASM170269v2, ASM14995v2, ASM1308505v1) obtained from the National Center for Biotechnology Information (NCBI) database (https://www.ncbi.nlm.nih.gov, accessed on 25 December 2024). The genome diagram was constructed using Circos v. 0.69.9 [32]. The genes and repeats were counted using Bowtie2 v. 2.5.3 [33] and bamCoverage v. 3.5.4 [34] for 50kb windows across the genome. Whole-genome alignment was performed using Nucmer v. 4.0.01 [35]. The entire analysis was conducted on the Galaxy platform [36].

Two approaches were used for identifying candidate effectors carried by *Forl* ZUM2407 and *Forc* V03-2g. The first approach was based on using the FoEC2 pipeline [37], which predicts candidate effector genes based on the *mimp* terminal inverted repeat (TIR) consensus sequence “TT[TA]TTGCNNCCCACTGNNN” [38]. The second approach was performed using a list of candidate effector genes taken from van Dam et al. [5]. The list based on the result of using the FoEC2 pipeline algorithm on 59 genomes of *Fox*, including formae speciales *radicis-cucumerinum* and *radicis-lycopersici* [5], was used as a reference list in this study. The alignment of reference candidate effector genes and the search for *mimp* TIRs in the *Forl* ZUM2407 and *Forc* V03-2g genomes were performed using UGene software packages v. 50 [39]. The *mimp* prediction was performed using the detection of two *mimp* TIRs in opposite directions within 400 bp of one another [37]. The genome of the non-pathogenic *Fusarium oxysporum* strain Fo47 (accession number: ASM1308505v1) was used as a reference to compare the profiles of the candidate effectors predicted in the *Forl* ZUM2407 and *Forc* V03-2g genomes.

##### Prediction of Candidate Effector Genes Associated with Cucumber Pathogenicity

For the prediction of common candidate effector genes associated with cucumber pathogenicity, the genomes of *Forc* V03-2g and *Forl* ZUM2407 were compared based on the following criteria: genes predicted in the vicinity of *mimp*, present in both *Forc* V03-2g and *Forl* ZUM2407, but absent in the non-pathogenic *Fox* Fo47 strain, should be candidates for virulence factors [16]; candidate effector genes located on chromosome 12 of *Forc* V03-2g, which shares synteny with the known pathogenicity chromosome (RC) of Forc016, are likely to play a role in cucumber pathogenesis [18].

#### 2.5.4. Phylogenetic Analysis

For phylogenetic analysis, the genomes of strains that share common host plants with *Forl* ZUM2407 and *Forc* V03-2g were used: *Foc* (*Fusarium oxysporum* f.sp. *cucumerinum*) strains 001, 011, and 013 (accession numbers: GCA_001702515.1, GCA_001703455.1, and GCA_001702495.1, respectively) causing wilt in cucumbers; *Fol* (*Fusarium oxysporum* f.sp. *lycopersici*) strains 069, 075, and 4287 (accession numbers: GCA_001703035.1, GCA_001703135.1, and GCA_000149955.2, respectively) causing wilt in tomatoes; *Forc* strains 016 and 024 causing shoot and root rot in cucumbers (accession numbers: GCA_001702695.2 and GCA_001702725.1, respectively); *Forl* CL57, causing shoot and root rot in tomatoes (accession number: GCA_000260155.1); and the non-pathogenic strain Fo47 (accession number: GCA_013085055.1) obtained from the National Center for Biotechnology Information (NCBI) database (https://www.ncbi.nlm.nih.gov accessed on 8 January 2025). Comparison was performed on concatenated genes *rpb1* (RNA polymerase II largest subunit), *rpb2* (RNA polymerase II second largest subunit), and *tef-1α* (translation elongation factor) using MAFFT v. 7.526 [40] (L-INS-i accuracy method) and IQTree. v. 2.3.6 [41] (maximum likelihood; bootstrap replicates = 1000). Sequences of these genes were predicted using alignments performed in UGene software [39] and extracted from the genomes of these strains. Comparison of the three candidate effectors was performed separately for each one using the same software. Trees were visualized with a phylogenetic tree viewer (https://beta.phylo.io accessed on 8 January 2025) without branch length with aligned tree leaves.

#### 2.5.5. Relative Gene Expression Analysis of Candidate Effectors in Forc V03-2g and Forl ZUM2407

Candidate effector gene expression in *Forc* V03-2g and *Forl* ZUM2407 was evaluated under laboratory conditions using both in vitro and in planta assays. For the in vitro assays, *Forc* V03-2g and *Forl* ZUM2407 were grown on PDA. Gene expression was evaluated after 7 days of growth. The in planta assay was caried out as described above in Section 2.3. For this purpose, the cucumber plant variety “Parizhskiy Kornishon”, susceptible to *Forc* V03-2g and *Forl* ZUM2407, was used. Gene expressions were evaluated after 2 and 14 days post-inoculation (dpi).

##### RNA Isolation and cDNA Synthesis

The total RNA was isolated from *Forc* V03-2g and *Forl* ZUM2407 mycelium for in vitro analysis and from 8 plants per replicate for in planta analysis and ground with liquid nitrogen using the ExtractRNA reagent (Evrogen, Moscow, Russia). The concentrations of isolated RNA were evaluated using a NanoDrop 2000C spectrophotometer (Thermo Fisher Scientific). The cDNA synthesis was performed using the MMLV RT kit (Evrogen, Moscow, Russian Federation) according to the manufacturer’s instructions. The final mixture included 1 μg total RNA and reverse primers of the corresponding candidate effector genes and β-tubulin, as a housekeeping gene for normalizing mRNA expression [42]. Primers were designed using CloneManager v.9 (Table S7).

##### qPCR Analysis

Gene expressions were quantitatively analyzed with the QuantStudio 5 Real-Time-PCR system (Thermo Fisher Scientific, USA) using the 5XqPCRmix-HS SYBR+LowROX kit (Evrogen, Moscow, Russian Federation). Reactions were carried out according to the manufacturer’s instructions. The thermal cycle conditions were set as follows: 3 min at 95 °C, followed by 40 cycles at 95 °C for 15 s and 60 °C for 60 s, and a melting curve stage consisting of 95 °C for 0.15 s, 60 °C for 1 min, and 95 °C for 0.1 s. Each sample was run thrice. The 2–ΔΔCT method of relative quantification was used to determine the fold change in expression [43].

## 3. Results

### 3.1. Disease Assay

**Figure 1:**
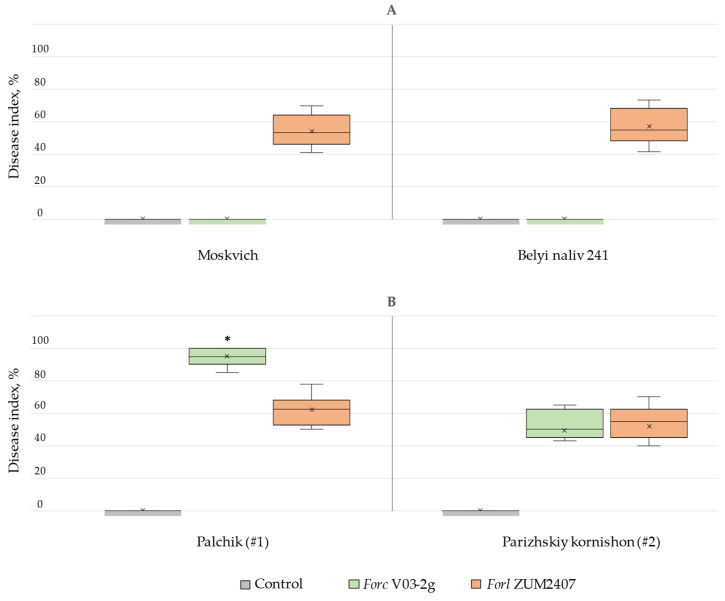
Disease indexes of (**A**) tomato varieties Moskvich and Beliy naliv 241, as well as (**B**) cucumber variety #1 (Palchik) and variety #2 (*Parizhskiy kornishon*), under treatment with *Forc* V03-2g and *Forl* ZUM2407. *—statistical difference among pretreated groups at *p* < 0.05.

After incubation, most cucumber plants developed typical foot and root rot disease symptoms with severe lesions. However, rots caused by *Forl* ZUM2407 and *Forc* V03-2g on cucumber plants spread both by ascending from the root system up the hypocotyl and by descending from the cotyledons as the disease assay was based on seed inoculation (Figures S1, S2 and S6d). The resulting disease indexes are shown in Figure 1.

The result indicated that *Forl* ZUM2407 caused root rot on tomato plants, whereas *Forc* V03-2g did not induce any symptoms compared to the untreated group (Figure 1A). The disease index of tomato plants pretreated with *Forl* ZUM2407 reached up to 54 ± 9% and 57 ± 10% for the Moskvich and Belyi naliv 241 varieties, respectively. In terms of their pathogenicity in cucumbers, it was observed that both strains, *Forl* ZUM2407 and *Forc* V03-2g, led to foot and root rot disease. The severity of disease recorded on plants depended on the plant varieties (Figure 1B). It was observed that, on cucumber plant variety #1, the rot caused by *Forl* ZUM2407 statistically differed from that induced by *Forc* V03-2g (Figure 1B). Most cucumber plants of variety #1 pretreated with *Forc* V03-2g died after 14 days post-inoculation. Meanwhile, cucumber plants of variety #1 pretreated with *Forl* ZUM2407 mainly displayed disease symptoms affecting the upper part of the hypocotyl (Figure S6d), although, in some plants, disease symptoms were similar to those of plants pretreated with *Forc* V03-2g (Figure S3). The disease indexes for cucumber variety #1 caused by *Forc* V03-2g and *Forl* ZUM2407 were assessed to be 95 ± 6% and 62 ± 9%, respectively.

In contrast, cucumber plant variety #2 exhibited a disease index of 49.6 ± 9% for *Forc* V03-2g and 52 ± 8% for *Forl* ZUM2407. The plant group pretreated with *Forc* V03-2g exhibited a four-fold higher number of dead plants compared with the group pretreated with *Forl* ZUM2407 (Figure S4). Meanwhile, other plants pretreated with *Forc* V03-2g exhibited a lower rot severity compared with those pretreated with *Forl* ZUM2407. Although the disease severity exhibited a different distribution rate, no statistically significant difference in disease index was observed between plants inoculated with *Forl* ZUM2407 and those inoculated with *Forc* V03-2g in cucumber variety #2. Furthermore, no statistically significant difference was found in the disease index between cucumber varieties #1 and #2 within the group pretreated with *Forl* ZUM2407.

To confirm the causal agents of the observed disease symptoms, single colonies were re-isolated from infected plant parts and subjected to BOX-PCR analysis to compare their genetic profiles. The obtained result is presented in Figures S1 and S3. All the selected isolates randomly generated an identical BOX profile.

### 3.2. Genome Assembly and Annotation

**Figure 2:**
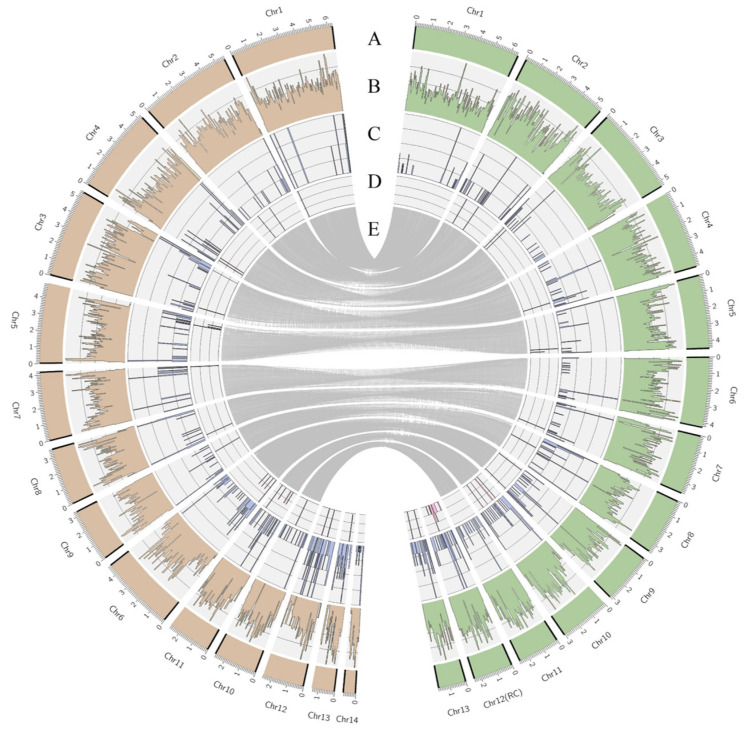
Diagram of *Forl* ZUM2407 (orange) and *Forc* V03-2g (green) genome assembly. (**A**) Chromosomes (black borders indicate the presence of telomeric repeats); (**B**) distribution of genes per chromosome; (**C**) distribution of repetitive regions; (**D**) distribution of candidate effectors indicated by van Dam et al. [5]; (**E**) gray lines show the core chromosome alignment between the two strains. The gene and repeat distributions in the figure are presented in relative units.

The whole-genome assembly of *Forc* V03-2g resulted in 20 contigs, 11 of which represent complete chromosomes, each flanked by telomeric repeats (CCCTAA) at both ends. Two contigs, Chr 4 and Chr 10, exhibit telomeric repeats on only one side. Notably, the assembly includes chromosome Chr 12, which displays a strong resemblance in size and synteny to the pathogenicity chromosome (RC) identified by van Dam et al. [8], except for an inverted central region (Figure S5). The genome assembly generated 13 chromosomes (Figure 2A) with a total size of 51.5 Mb. Among these, 11 represent the core chromosomes of *Forc*, aligning with the number found in other *Fox* strains. The remaining two chromosomes constitute the accessory genome specific to strain *Forc* V03-2g.

The whole-genome assembly of *Forl* ZUM2407 generated 35 contigs, 9 of which represent full-length chromosomes with telomeric repeats at both ends, including a 785 kbp contig (Chr14). Six additional contigs displayed telomeric repeats at one end, two of which also contained ribosomal DNA repeats at their termini. These contigs were subsequently merged to form chromosome number 3. The total assembly generated 14 chromosomes (Figure 2A), 11 of which represent the general core chromosomes homologous to *Forc* V03-2g chromosomes (Figure 2E), with 3 chromosomes representing the accessory genomes of *Forl* ZUM2407. The genome assembly was deposited in the NCBI genome database under BioProject ID: PRJNA1186086 (accessed on 4 December 2024).

The accessory genomes of *Forc* V03-2g and *Forl* ZUM2407, consisting of two and three chromosomes, respectively, exhibit no synteny with each other. All contigs comprising the accessory genome exhibit a higher repeat content and lower gene density compared with the core genome (Figure 2B,C). Notably, repeat-rich regions with a low gene density are also observed within the core chromosomes of *Forl* ZUM2407, specifically at the flanks of chromosomes 3 and 6 (Figure 2C).

**Figure 3:**
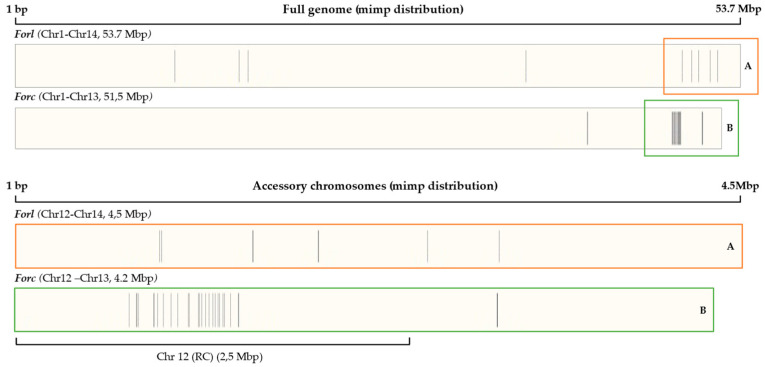
Distribution of *mimp*s (represented by gray lines) in the genomes of the *Forc* V03-2g and *Forl* ZUM2407 strains. (**A**) Part of the *Forl* genome and (**B**) part of the *Forc* genome represented by accessory chromosomes.

The number of *mimp*s in the *Forl* ZUM2407 whole genome is three times lower than that of *Forc* V03-2g (10 and 36, respectively), with almost six-fold less accessory chromosomes (6 and 34, respectively). The *mimp*s in the *Forl* ZUM2407 genome are not clustered as predicted for the *Forc* V03-2g pathogenicity chromosome Chr12 (RC), which accounts for 32 *mimp*s (Figure 3A), while all *Forl* ZUM2407 accessory chromosomes account for 6 *mimp*s (Figure 3B).

### 3.3. Candidate Effector Profile

Using the FoEC2 pipeline without the reference candidate effectors, 5 and 22 genes were predicted in the genomes of *Forl* ZUM2407 and *Forc* V03-2g, respectively. Among the predicted genes, there were no candidate effectors common to both strains which were localized on the accessory chromosomes and absent in the non-pathogenic strain Fo47. Most of the predicted genes were present in the list of 104 reference effectors [5].

Among the reference effectors, we identified 50 genes with a nucleotide similarity of more than 70% in the *Forc* V03-2g and *Forl* ZUM2407 genomes, which included 38 genes with a nucleotide similarity of more than 95% (Figure 2D). Among the 50 genes predicted by aligning the reference list, common candidate effectors to both strains were identified. Approximately half of the reference candidate effector genes predicted in the genomes of *Forl* ZUM2407 and *Forc* V03-2g were not located near *mimp* transposons. Consequently, these genes could not be identified in these strains using a *mimp*-based approach. The predicted candidate effector genes in *Forc* V03-2g and *Forl* ZUM2407 are listed in Table 1.

### 3.4. Prediction of Common Candidate Effector Genes Associated with Cucumber Pathogenicity

According to previous findings reported by Li et al. [8], most effector genes, including *SIX*6, *SIX*9, *SIX*11, and *SIX*13, are located on chromosome RC. However, *Forl* ZUM2407 lacks 15 well-known *SIX* genes or their homologs that are present in *Fox.*

However, three common candidate effector genes, metallo-beta-lactamase family protein, carbonic anhydrase, and malate dehydrogenase, predicted in both the *Forc* V03-2g and *Forl* ZUM2407 genomes were absent in strain Fo47. The metallo-beta-lactamase family protein, exhibiting 99.7% and 98.6% nucleotide similarity to the reference candidate effector in *Forc* V03-2g and *Forl* ZUM2407, respectively, was located within their core chromosomes and was not associated with *mimp* in both genomes. Two notable genes, carbonic anhydrase (with 100% and 79.9% similarity to the reference candidate effector in *Forc* V03-2g and *Forl* ZUM2407, respectively) and malate dehydrogenase (with 84.3% and 99.5% similarity, respectively), were both located on their accessory chromosomes, particularly on Chr 12 (RC) in the Forc V03-2g genome (Table 1).

The analysis of common predicted genes in relation to their association with *mimp* revealed notable differences between the genomes. The *carbonic anhydrase* gene in *Forl* ZUM2407, in contrast to its homolog in *Forc* V03-2g, is not associated with *mimp*, and the malate dehydrogenase gene in *Forc* V03-2g, in contrast to its homolog in *Forl* ZUM2407, is also not associated with *mimp*. Considering only the genes associated with *mimp* in these strains, *Forc* V03-2g and *Forl* ZUM2407 do not have any common candidate effectors.

Additionally, the gene *FOMG_17360* present in the non-pathogenic strain Fo47 with identical nucleotides to the reference probably cannot be considered an independent marker of pathogenicity.

### 3.5. Phylogenetic Trees

#### 3.5.1. Phylogenetic Tree Based on Concatenated *rpb1*, *rpb2*, and *tef1-α* Genes

Phylogenetic analysis was carried out to clarify the position of the *Forc* V03-2g and *Forl* ZUM2407 strains among other close *Fox* formae speciales. A phylogenetic tree was constructed for the strains capable of causing disease in tomato and cucumber plants, including the non-pathogenic strain Fo47.

**Figure 4:**
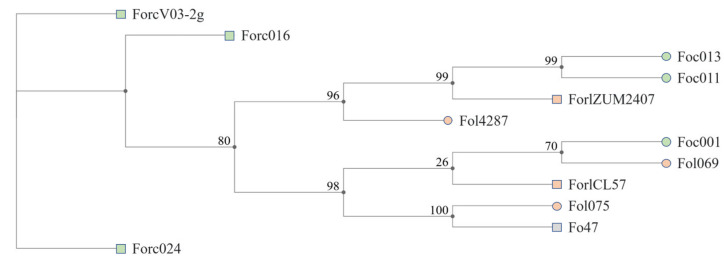
Maximum likelihood phylogenetic tree of 12 Fox strains, based on concatenated *rpb1* (5362 nt), *rpb2* (3907 nt), and *tef-1α* (1772 nt) genes (nt—nucleotides). Squares indicate strains causing foot and root rots; circles indicate strains causing wilt; green color—pathogens of cucumbers; orange—pathogens of tomatoes; gray square—non-pathogenic strain Fo47. Gene sequences are presented in Table S7.

As shown in Figure 4, those strains are grouped independently of their ability to cause disease and host specificity. The *Forl* ZUM207 and *Forc* V03-2g strains do not belong to the same clade; meanwhile, the non-pathogenic strain Fo47 belongs to the clade with the wilt pathogen *Fol*075.

#### 3.5.2. Phylogenetic Tree Based on Common Candidate Effector Genes

**Figure 5:**
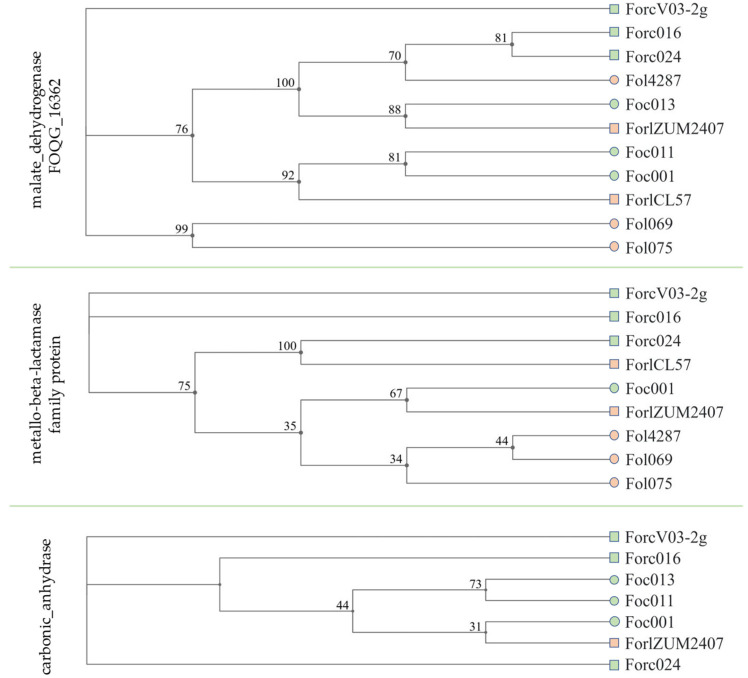
Maximum likelihood phylogenetic tree of 12 *Fox* strains, based on malate dehydrogenase, metallo-beta-lactamase family protein, and carbonic anhydrase genes, listed in Table 1. Squares indicate strains causing foot and root rots; circles indicate strains causing wilt; green color—pathogens of cucumbers; orange—pathogens of tomatoes. Strains that are not included in the trees do not have these genes in their genomes. Genes sequences are presented in Table S7.

Three common genes (malate dehydrogenase, metallo-beta-lactamase family protein, carbonic anhydrase) predicted in the *Forl* ZUM207 and *Forc* V03-2g genomes were tested in terms of their presence in strains with similar host plants (Figure 5).

The phylogenetic tree based on the malate dehydrogenase gene is characterized by the proximity of the *Forc*016 and *Forc*024 strains to the *Fol*4287 strain, which has not been described as a pathogen in cucumbers, which probably excludes the relation of this gene to the pathogenesis in cucumbers.

The phylogenetic tree based on the metallo-beta-lactamase family protein gene places *Fol* strains in a separate clade to the pathogens of cucumbers (*Foc*001, *Forl* ZUM2407, and *Forc* strains); however, two out of the three considered *Foc* strains do not have this gene in their genomes and consequently are not included in this tree. In this regard, this gene can probably be excluded as optional for formae speciales causing disease in cucumbers.

The phylogenetic tree based on the carbonic anhydrase gene includes only strains capable of causing disease in cucumber plants among the 12 *Fox* strains used. However, for all *Forc* and *Foc* strains, the nucleotide similarity to the reference sequence is greater than 99%, while for *Forl* ZUM2407, this similarity is 79.9%.

### 3.6. Common Candidate Effector Gene Expression Analysis

**Figure 6:**
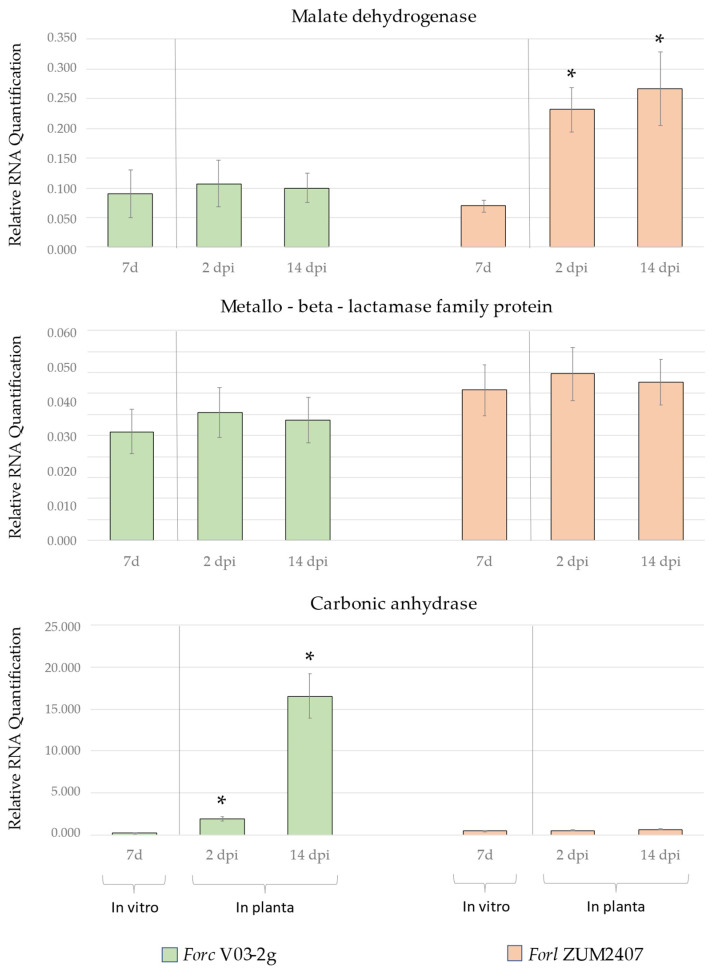
Relative mRNA quantification (RQ) of common candidate effector genes predicted in *Forc* V03-2g and *Forl* ZUM2407. For the in vitro assay, the relative expression was assessed after a 7-day incubation on PDA medium. Meanwhile, for the in planta assay, the relative expression of common candidate effector genes was evaluated after 2 and 14 dpi of cucumber plants by the *Forc* V03-2g and *Forl* ZUM2407 strains. *—indicates genes whose RQ in planta statistically differed from its in vitro expression at *p* ≤ 0.05.

The relative gene expressions of the common predicted candidate effectors in *Forc* V03-2g and *Forl* ZUM2407 were analyzed, and the obtained result is shown in Figure 6. Malate dehydrogenase gene expression in Forc V03-2g remained consistent under in planta and in vitro conditions, showing a low relative expression level. In contrast, Forl ZUM2407, which showed a low level of malate dehydrogenase expression in vitro, increased after cucumber plant inoculation, with similar levels (at *p* ≤ 0.05) observed at both 2 and 14 dpi. The relative gene expression of the metallo-beta-lactamase family protein gene remained consistent in both the *Forc* V03-2g and *Forl* ZUM2407 strains under both in vitro and in planta conditions (2 and 14 dpi).

In contrast, the relative carbonic anhydrase gene expression in *Forc* V03-2g increased significantly after 2 dpi compared with the in vitro levels and further increased after 14 dpi, when foot and root rot symptoms were clearly observed. Meanwhile, the *Forl* ZUM2407 homolog of this gene did not exhibit this expression pattern and had a low expression under in planta and in vitro conditions.

## 4. Discussion

Several studies have reported the ability of *Forl* to cause root rot in both tomato and cucumber plants when inoculated through the seeds or roots [44,45,46]. While root rot is the primary symptom observed in these studies, seed inoculation can also lead to damping-off, as reported by other researchers [46]. In our study, we observed differences in the severity of disease symptoms caused by *Forc* V03-2g in different cucumber varieties. However, these differences do not appear to be indicative of distinct races within this forma specialis [47], as disease symptoms were observed in all tested cucumber plant varieties. Conversely, *Forl* ZUM2407 did not exhibit significant differences in disease severity between the two cucumber varieties.

Genomic analysis of *Forl* ZUM2407 with the pathogenic chromosome (RC) of *Forc* V03-2g did not predict any syntenic regions, in contrast to other formae speciales such as *Fox* f.sp. *melonis*, which shares a common host plant (melon) with *Forc* [48]. *SIX* gene sequences have not been found in either the assembly or reads of the *Forl* ZUM2407 genome. The absence of these genes in pathogenic root rot-causing strains of *Fox* is not rare. Other authors also point to their absence in pathogenic *Forl* strains [5,49,50] and in *Fox* strains causing root rot in peas [20], as well as in *Fox* strains causing bulb rot in onions [19]. Given the non-homologous nature of these genes with each other and their possible association with pathogens that cause wilting rather than root rot, we assume that not each of them is related to root rot pathogenesis, at least in cucumber, as demonstrated by the deletion of the *SIX*9 gene in strain *Forc*016, which showed no change in the disease index in the whole list of plants (cucumber, melon, watermelon) characteristic to this forma specialis [8]. Meanwhile, the deletion of the *SIX*6 gene in the work of the same authors showed a decrease in the aggressiveness of the pathogen towards cucumbers at temperatures above 25 °C but did not lead to a complete loss of pathogenicity in these plants. This gene could likely be one of the reasonable explanations for the difference in the rate of root rot development in cucumber between *Forl* ZUM2407 and *Forc* V03-2g.

The phylogenetic tree based on the core genes (*rpb1*, *rpb2*, and *tef1-α*) shows *Forl* ZUM2407 forming a clade with two cucumber pathogens (*Foc*013 and *Foc*011), as well as *Forl*CL57 reported in the previous work of van Dam et al. [5] forming a clade with one tomato pathogen (*Fol*069) and one cucumber pathogen (*Foc*001), attesting to the polyphyletic origin of the two *Forl* strains. Interestingly, the malate dehydrogenase and metallo-beta-lactamase family protein genes are present in both tomato and cucumber pathogens, but carbonic anhydrase is related only to cucumber pathogens, including *Forl* ZUM2407. Also, carbonic anhydrase was hypothesized to be a pathogenesis-related gene in *Fusarium virguliform* causing sudden death syndrome in soybean plants [51], which may suggest its role in pathogenesis for *Fox* strains.

Expression analysis of candidate effector genes revealed distinct patterns for *Forc* V03-2g and *Forl* ZUM2407. Carbonic anhydrase expression in Forc V03-2g showed the highest RQ value when foot and root rot symptoms on cucumber were clearly observed, supporting the hypothesis that this gene functions as an effector [5]. Conversely, malate dehydrogenase expression was elevated in *Forl* ZUM2407, while carbonic anhydrase and the metallo-beta-lactamase family protein gene remained unchanged. However, the increase in malate dehydrogenase expression in *Forl* ZUM2407 was significantly less than that of the carbonic anhydrase expression observed in *Forc* V03-2g and did not correlate with the emerging foot and root rot symptoms in cucumbers. Therefore, malate dehydrogenase is unlikely to be involved in cucumber pathogenesis. The metallo-beta-lactamase family protein gene showed a lack of gene expression change in both strains after 2 and 14 dpi under in planta conditions, which may suggest that it is not involved in cucumber pathogenesis.

Furthermore, no common effectors with a higher expression in planta were identified between the two strains. In this regard, we propose that the unique candidate effector genes in *Forl* ZUM2407 identified using the *mimp*-based approach or other genes that cannot be predicted by this method and have nothing in common with *Forc* V03-2g effectors may play a role in the pathogenesis in cucumber plants, which can be determined through transcriptome analysis.

Overall, we suggest that the presence of *mimp* transposons in the vicinity of the gene is more an indicator of the organization of a particular type of chromosome subjected to horizontal transfer [52], rather than a guaranteed indicator of an effector in any *Fox* formae speciales, especially in strains causing root rot. This assumption is consistent with the analysis of effector candidates shown in Table 1, primarily because of the absence of *SIX* genes in *Forl* ZUM2407; based on their association with the *mimp* mobile element, the candidate effector search algorithm for *Fox* was built. In addition, the results of the low expression in planta of the *malate dehydrogenase* gene associated with *mimp* in *Forl* ZUM2407 and the *metallo-beta-lactamase family protein* gene having a high nucleotide similarity (Table 1) to the reference candidate effector gene associated with *mimp* also support this assumption. However, the differences in the rate of disease development between strains in cucumber variety #1 suggest that the pathogenicity gene sets in *Forc* V03-2g mediate disease development in this crop more effectively than the gene set in *Forl* ZUM2407.

## 5. Conclusions

Although *Forl* ZUM2407 is a tomato pathogen, it was able to infect cucumber plants, showing the broader range of its host plants. The accessory genomes of both strains are not syntenic with each other, and the number of *mimp*s on all the accessory chromosomes is lower in *Forl* ZUM2407 than in *Forc* V03-2g on chromosome RC by almost five-fold (6 and 32, respectively). Since *Forl* ZUM2407 causes foot and root rot in tomato and cucumber plants without having any of the *SIX* genes and their homologs, there might be other effector genes responsible for root rot development in this strain. Also, since the in silico analysis was not able to predict common effector genes associated with *mimp* transposons in *Forl* ZUM2407 and *Forc* V03-2g, we suggest that transcriptome analysis may help to predict effector genes that might be responsible for the pathogenicity of these strains in cucumber plants.

## Figures and Tables

**Table 1:** List of candidate effector genes from the list by van Dam et al. [5] that are present in the *Forc* V03-2g and *Forl* ZUM2407 genomes.

Candidate Effectors (blastX Annotation)	*Forc*	*Forl*	Location *Forc/Forl*	Fo47	Per. Ident to Ref. Fo47	*mimp*s *Forc/Forl*	Per. Ident to Ref. *Forc/Forl*
Accessory Chromosomes							
SIX6	+	−	Chr12 (RC)	−	−	+	100
SIX9	+	−	Chr12 (RC)	−	−	+	100
SIX11	+	−	Chr12 (RC)	−	−	+	100
SIX13	+	−	Chr12 (RC)	−	−	+	100
FomEff1a	+	−	Chr12 (RC)	−	−	+	100
Endopolygalacturonase	+	−	Chr12 (RC)	−	−	+	90
Metalloendopeptidase	+	−	Chr12 (RC)	−	−	+	97.1
Pep1-like protein	+	−	Chr12 (RC)	−	−	+	99
Npp1 domain protein	+	−	Chr12 (RC)	−	−	+	100
Early nodulin 75 precursor	+	−	Chr12 (RC)	−	−	−	94.4
Hypothetical protein FOMG_19810	+	−	Chr12 (RC)	−	−	−	100
Hypothetical protein FOPG_18590	+	−	Chr12 (RC)	−	−	−	96.6
Hypothetical protein FOTG_17952	+	−	Chr12 (RC)	−	−	−	94.1
Unknown	+	−	Chr12 (RC)	−	−	+	91.6
Hypothetical protein FOMG_20013	+	−	Chr12 (RC)	−	−	+	100
Hypothetical protein FOMG_19517	+	−	Chr12 (RC)	−	−	−	100
Hypothetical protein AU210_015946	+	−	Chr12 (RC)	−	−	+	100
Hypothetical protein FOMG_18485	+		Chr12 (RC)	−	−	+	100
Catalase-peroxidase2	+		Chr12 (RC)	−	−	−	95.3
Oxidoreductase	+	−	Chr12 (RC)	−	−	−	96.3
Unknown	−	+	Chr13	−	−	+	100
Proteasekex1	+	−	Chr12 (RC)	+	98.8	−	98.9
Carbonic anhydrase *	+	+	Chr12 (RC)/Chr12	−	−	+/−	100/79.9
Hypothetical protein FOIG_09834	+	+	Chr12 (RC)/Chr12	+	98.5	−	98.5/98.6
Malate dehydrogenase FOQG_16362 *	+	+	Chr12 (RC)/Chr12	−	−	−/+	84.3/99.5
Hypothetical protein BFJ65_g18369	+	+	Chr12 (RC)/Chr13	+	90.3	−/−	90.3/89.6
Core chromosomes							
Glycoside hydrolase family 43	+	−	Chr9	+	99.2	−	99.1
Fad binding domain-containing protein	−	+	Chr6	−	−	+	99.3
Hypothetical protein FOMG_17360	−	+	Chr3	+	100	−	98.4
Hypothetical protein FOQG_18109	−	+	Chr6	−	−	−	91.3
Alphamannosidase	−	+	Chr4	−	−	−	99.7
Metallo-beta-lactamase family protein *	+	+	Chr3/Chr4	−	−	−/−	99.7/98.6
Catalase-peroxidase_2	+	+	Chr11/Chr10	+	79.4	−/−	79.9/74.2
Oxidoreductase	+	+	Chr9/Chr6	+	80.1	−/−	81.1/80.1
Proteasekex1	+	+	Chr4/Chr3	+	98.8	−/−	98.9/99
Others	+	+		+		−/−	

N.B.: Green color indicates genes that are located only in the *Forc* V03-2g genome. Orange indicates genes that are located only in the *Forl* ZUM2407 genome. In gray are those located in both strains. “*” indicates the common candidate effector genes in both genomes that are absent in strain Fo47. “+” means gene/*mimp* presence; “−” means gene/*mimp* absence. The full table with genomic sequences is presented in Table S7.

## Data Availability

The original contributions presented in the study are included in the article and Supplementary Materials; further inquiries can be directed to the corresponding authors.

## Supplementary Materials

The following supporting information can be downloaded at: https://www.mdpi.com/article/10.3390/jof11020140/s1, Figures S1, S2, S3, S4, S5, S6a-d, Table S7.
