# Supplementary material for: Genomic Differences Between Two Fusarium oxysporum Formae Speciales Causing Root Rot in Cucumber
Source: J Fungi (Basel). 2025 Feb 12;11(2):140. doi: 10.3390/jof11020140 (PMC11856433; doi:10.3390/jof11020140)
Supplement: Supplementary file 1 [file jof-11-00140-s001.zip › Supplementary file 1. Figures S1-S6d.pdf]

# Genomic Differences between Two *Fusarium oxysporum* Formae Speciales Causing Root Rots in Cucumber

Ernest Nailevich Komissarov[1]*, Roderic Gilles Claret Diabankana[1]*, Inna Abdeeva[2], Daniel Mawuena Afordoanyi[1], Sergey Vladimirovich Gudkov[3,4], Ekaterina Mikhailovna Dvorianinova[5], Sergey Alexandrovich Bruskin[2], Alexey Alexandrovich Dmitriev[5] and Shamil Zavdatovich Validov[1]

1 Laboratory of Molecular Genetics and Microbiology Methods, Kazan Scientific Center of the Russian Academy of Sciences, 420111 Kazan, Russia;
2 Vavilov Institute of General Genetics Russian Academy of Sciences, 119991, Moscow, Russia
3 Prokhorov General Physics Institute of Russian Academy of Sciences, 119991 Moscow, Russia
4 Institute of Biology and Biomedicine, Lobachevsky State University of Nizhny Novgorod, 603022 Nizhny Novgorod, Russia
5 Engelhardt Institute of Molecular Biology, Russian Academy of Sciences, 119991 Moscow, Russia
* Correspondence: e.komissarov@knc.ru, r.diabankana@knc.ru

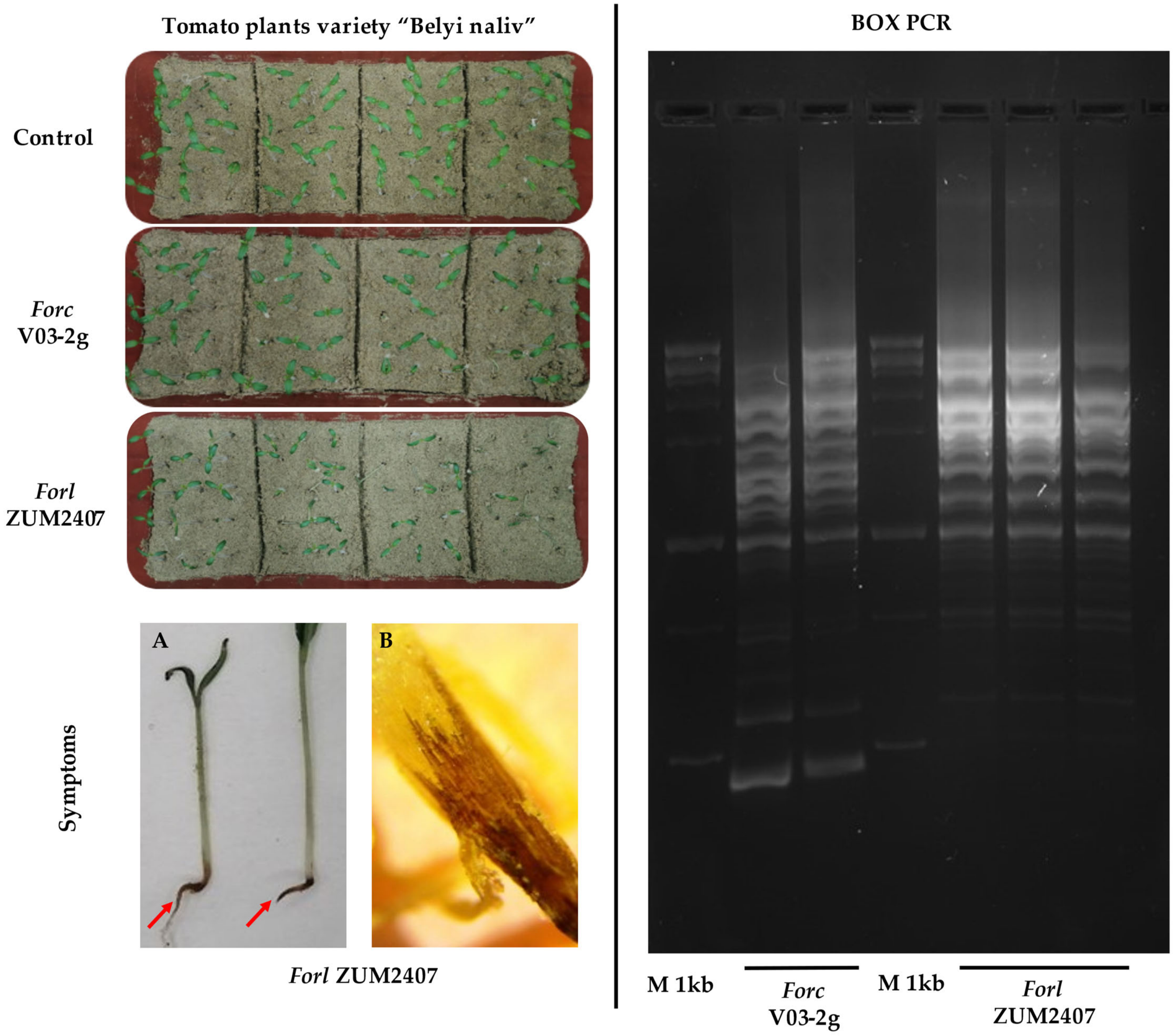

**Figure S1.** Tomato disease assay and symptoms caused by *Forl* ZUM2407 on tomato roots. *Forc* V03-2g and *Forl* ZUM2407 BOX PCR analysis. (red arrows point at root rots/ "M" – means marker: 1 kb ladder)

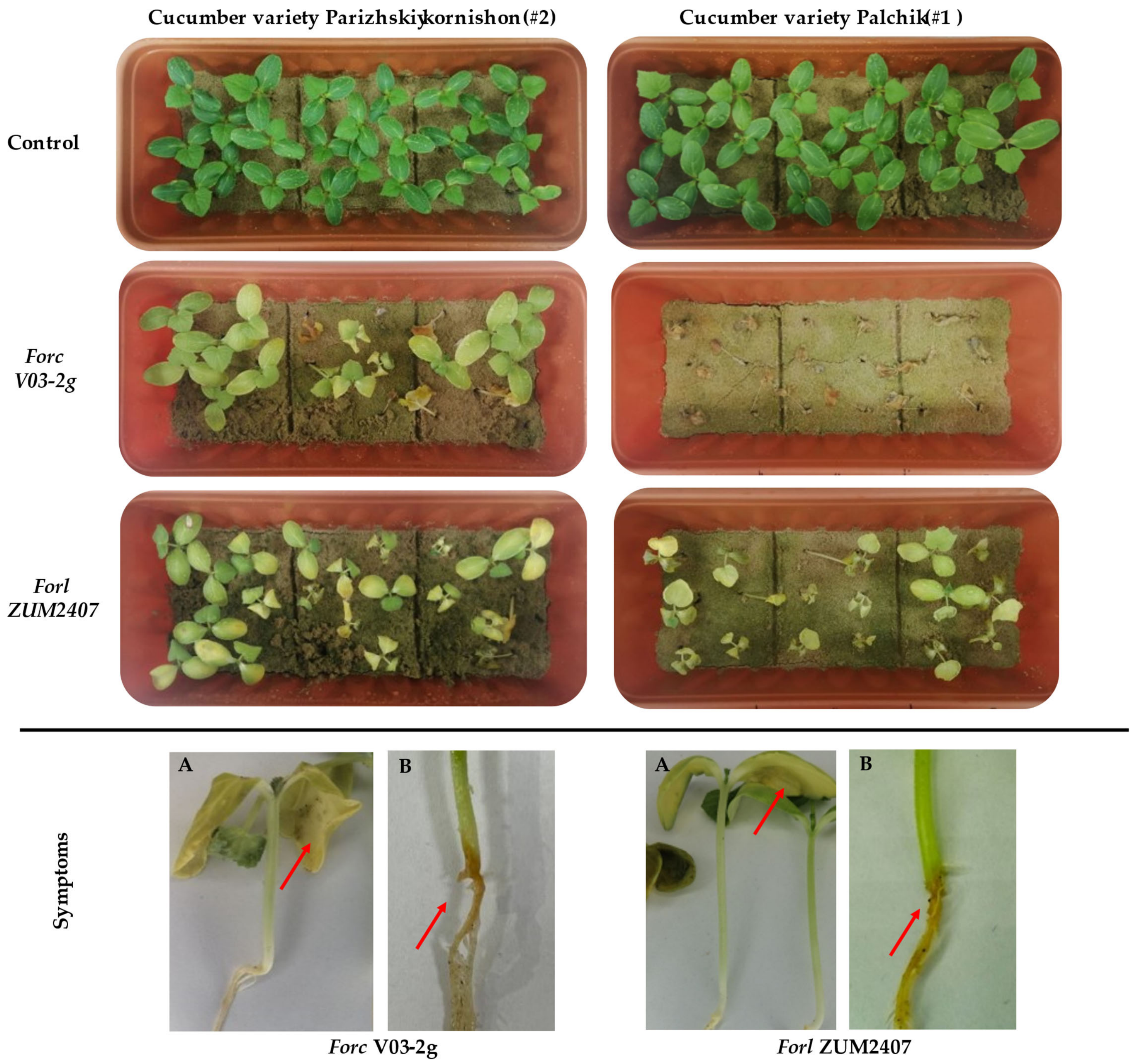


**Figure S2.** Disease assay on two cucumber varieties and symptoms caused by *Forc* V03-2g and *Forl* ZUM2407 strains [red arrows point at damping off (A) and root rots (B)]

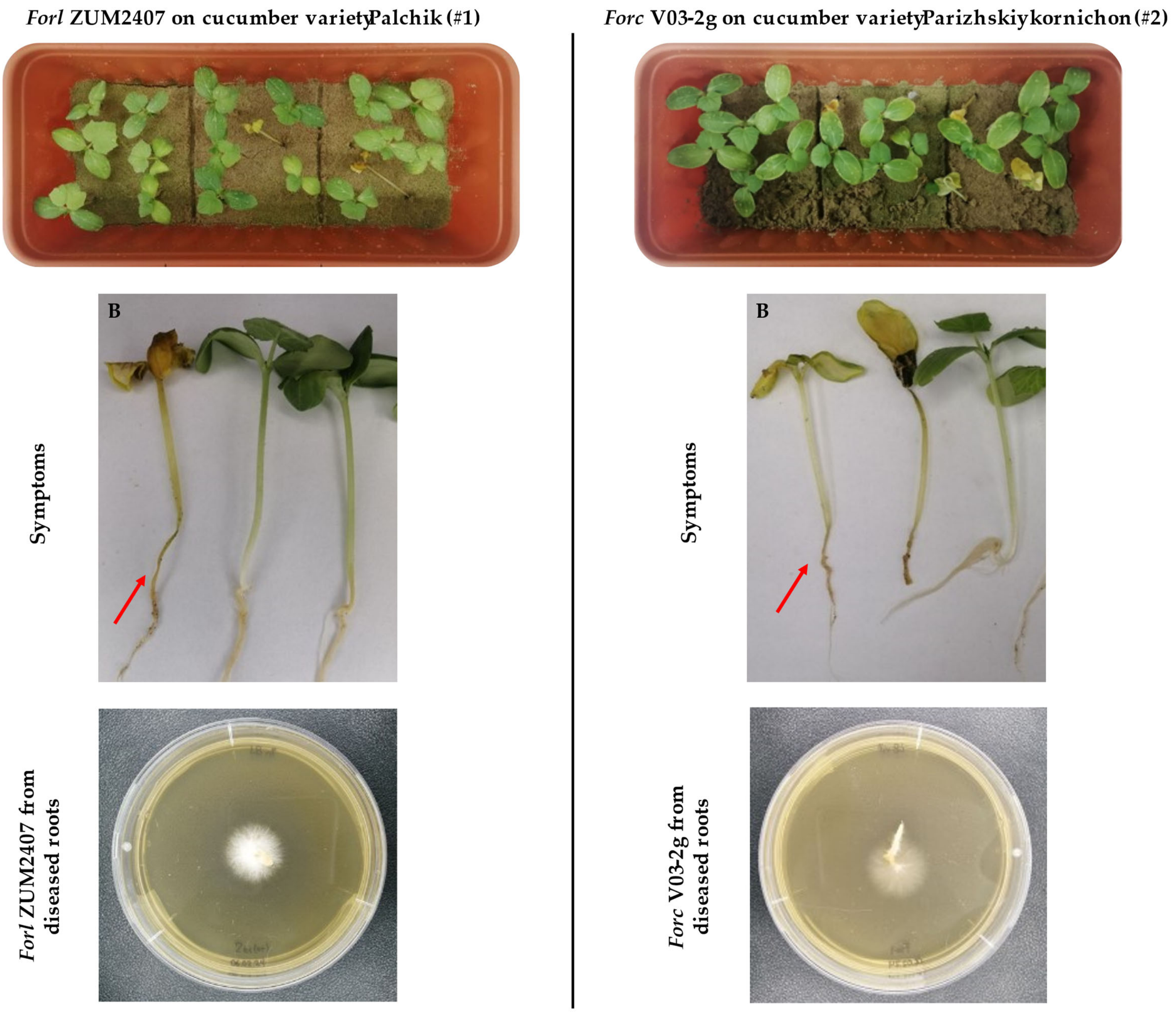


**Figure S3.** Disease development on cucumber plants between strains *Forc* V03-2g on variety Parizhskiy kornichon (#2) and *Forl* ZUM2407 on variety Palchik (#1). Reisolation from cucumber affected areas. [red arrows point at root and foot rots (B)]

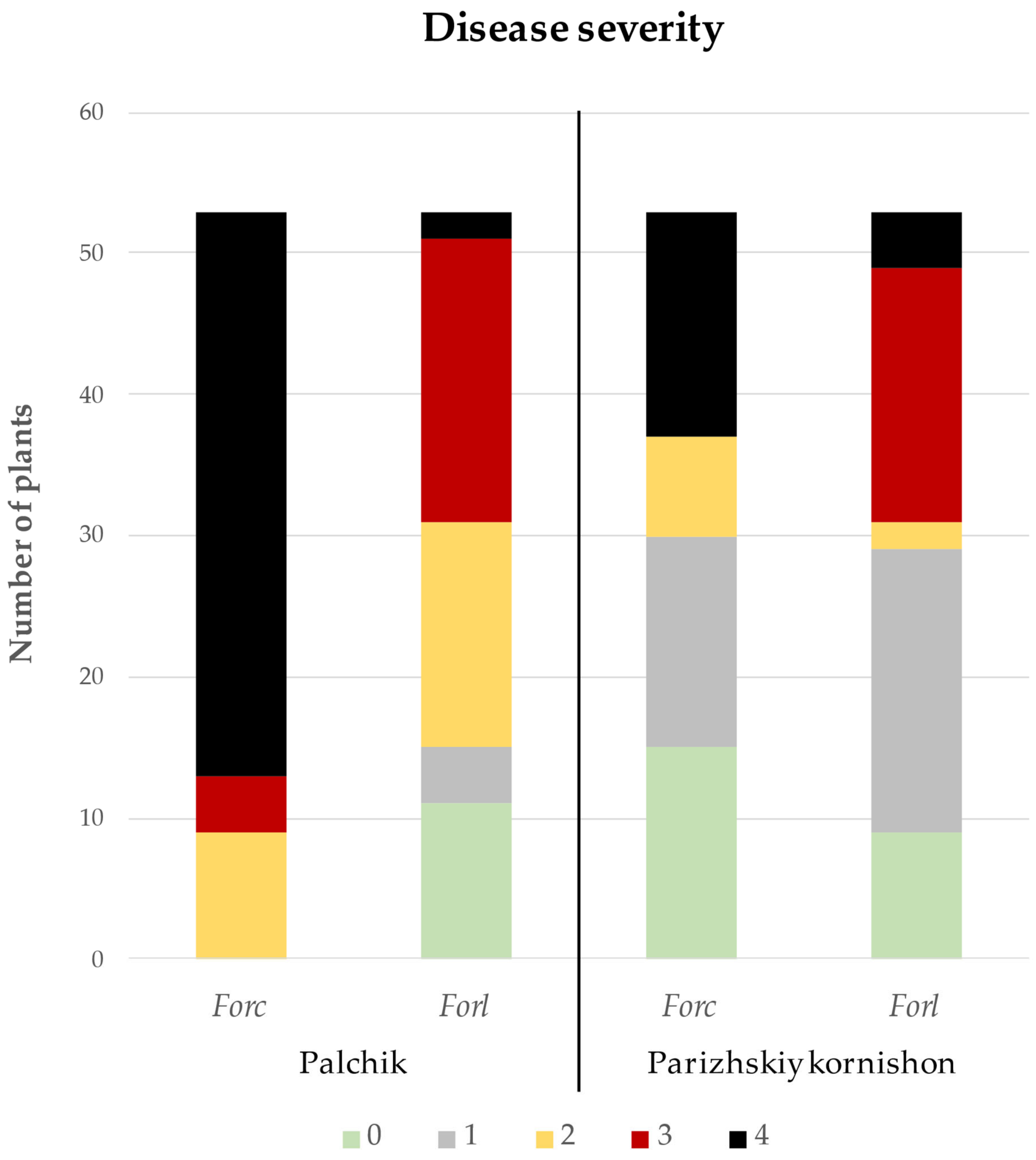


**Figure S4.** Disease severity on two cucumber varieties pretreated with *Forc* V03-2g and *Forl* ZUM2407 strains. Assessment was based on 0-4 scale*: 0 – healthy plants; 1 - plants with small lesions (weak roots browning/weak cotyledons chlorosis); 2 - plants with developed lesions (clearly visible symptoms of root rot in the area of root neck and main root/ yellowed cotyledons); 3 - plants with large lesions (root rot ascends along hypocotyl to cotyledons/ wilted and browned cotyledons); 4 - dead plants (Figure S6.1-S6.4)*

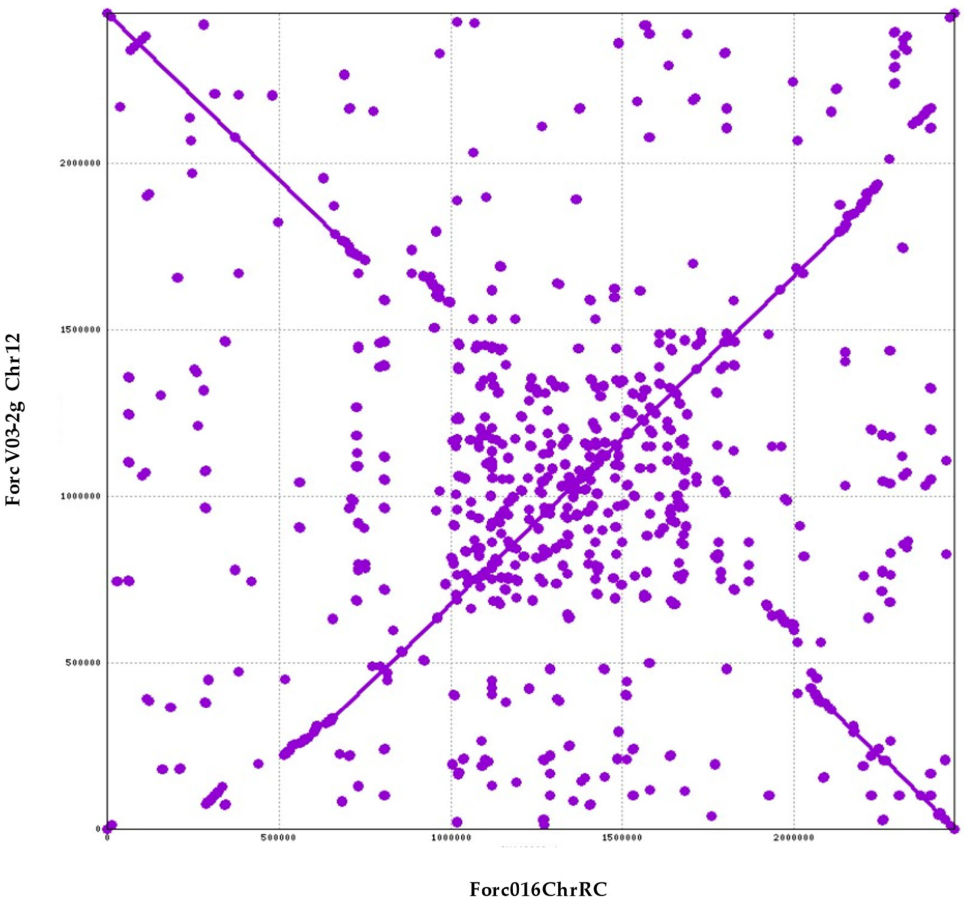


**Figure S5.** Similarity between *Forc* V03-2g chromosome 12 and the *Forc*016 pathogenicity chromosome RC.

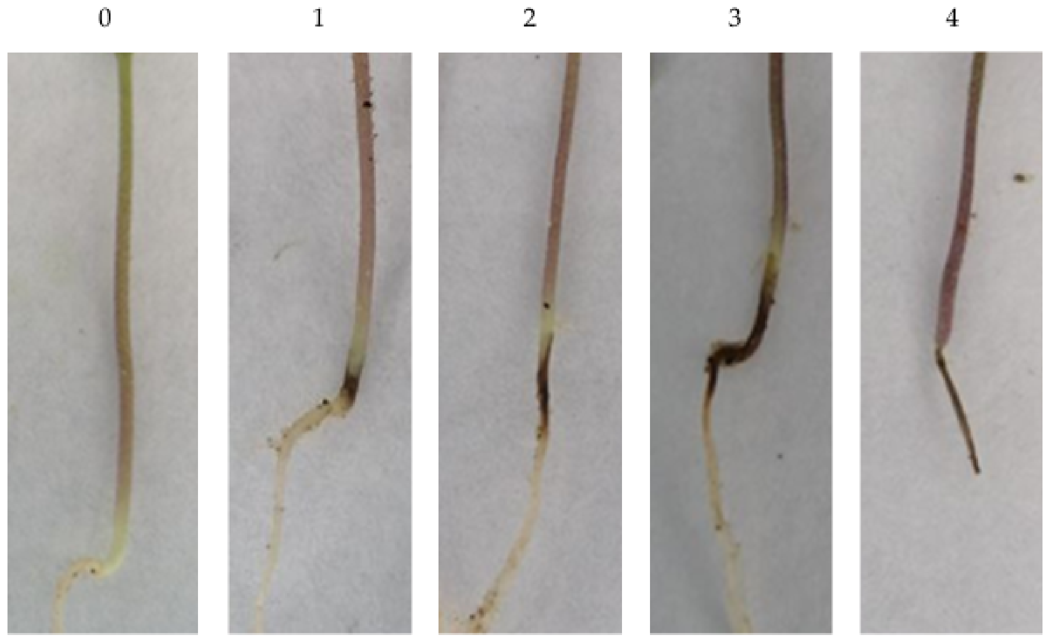


**Figure S6a.** Foot and root rot on tomato plants. Severity symptom scale (0-4).

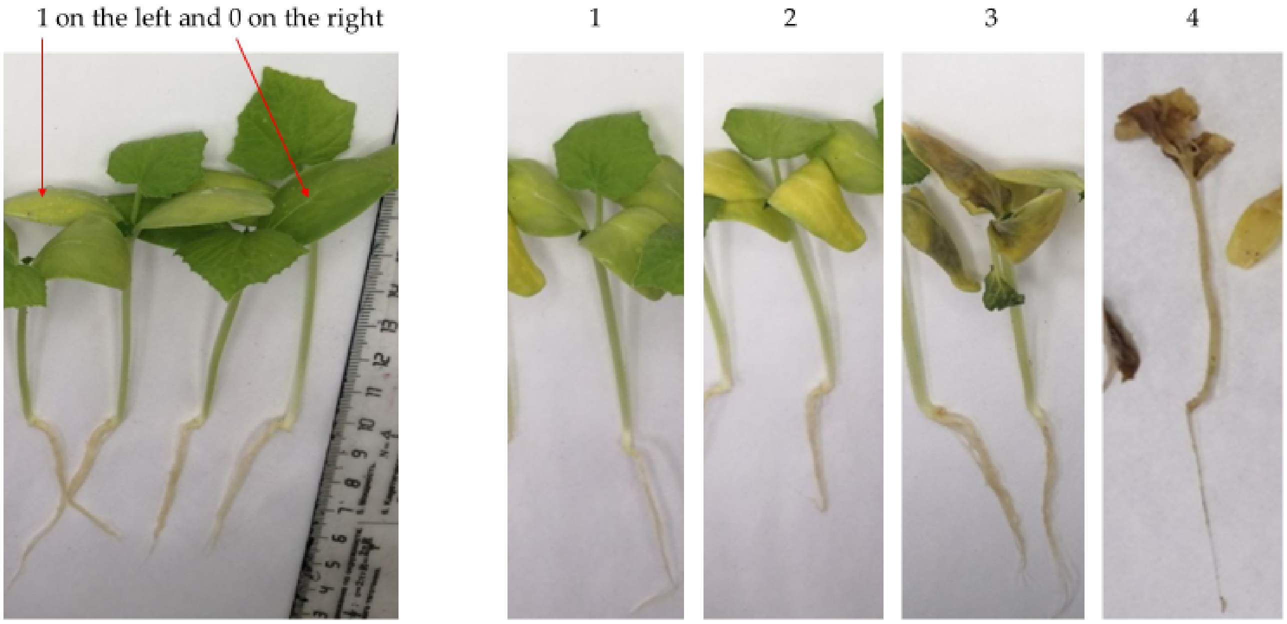


**Figure S6b.** Damping off on cucumber plants. Severity symptom scale (0-4).

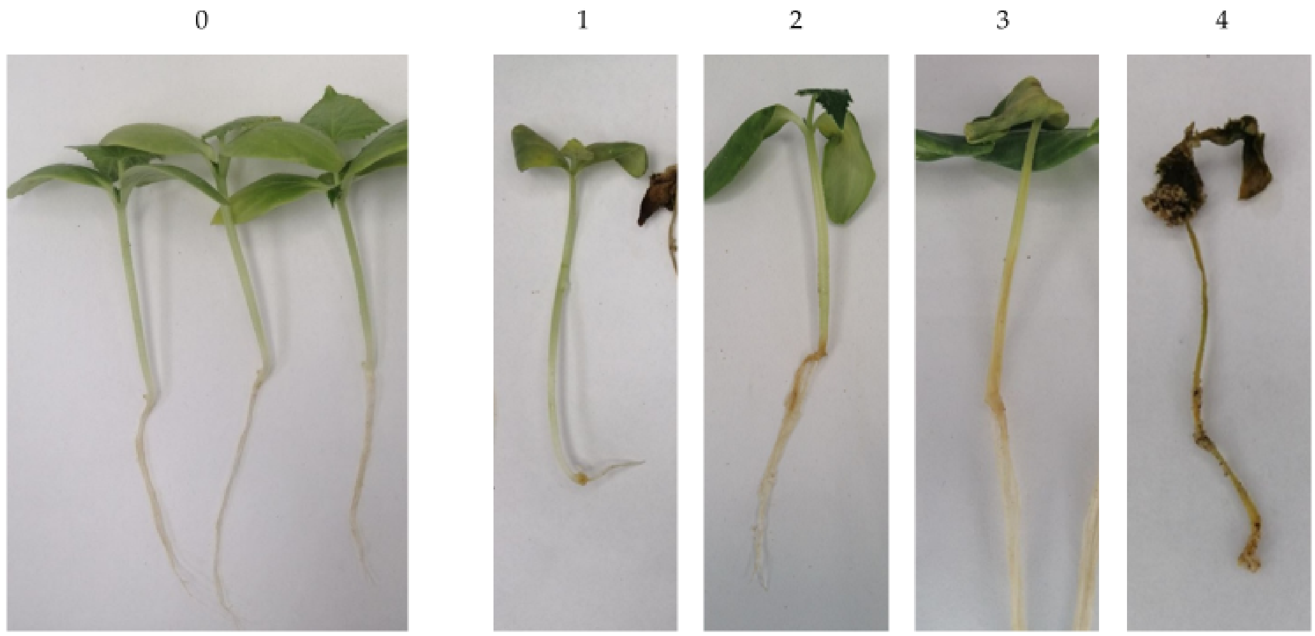


**Figure S6c.** Foot and root rot on cucumber plants. Severity symptom scale (0-4).

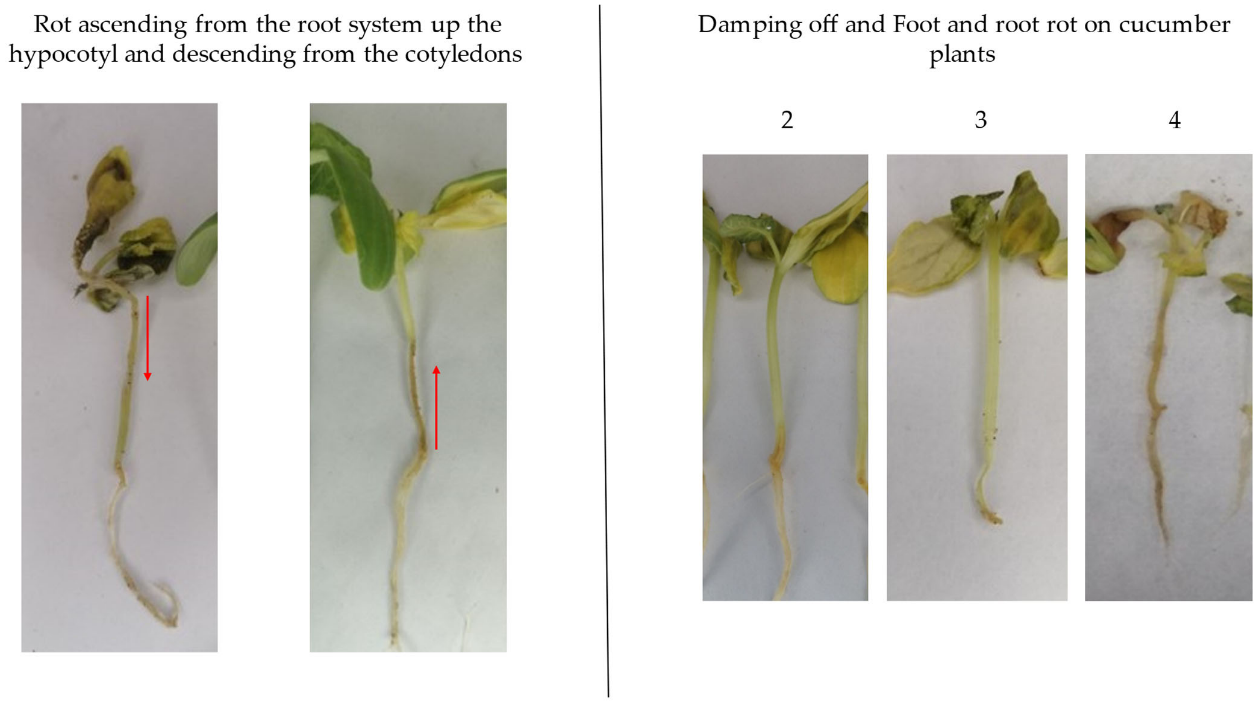


**Figure S6d.** Damping off and foot and root rot on cucumber plants. Severity symptom scale (0-4).
